# Ptk7 Is Dynamically Localized at Neural Crest Cell–Cell Contact Sites and Functions in Contact Inhibition of Locomotion

**DOI:** 10.3390/ijms22179324

**Published:** 2021-08-28

**Authors:** Anita Grund, Katharina Till, Klaudia Giehl, Annette Borchers

**Affiliations:** 1 Faculty of Biology, Molecular Embryology, Philipps-University Marburg, D-35032 Marburg, Germany; anita-grund@outlook.com (A.G.); tillka@staff.uni-marburg.de (K.T.); 2 Faculty of Medicine, Signal Transduction of Cellular Motility, Internal Medicine V, Justus-Liebig University Giessen, D-35392 Giessen, Germany; Klaudia.Giehl@innere.med.uni-giessen.de

**Keywords:** neural crest migration, PTK7, dynamic cell–cell contacts, cell invasion

## Abstract

Neural crest (NC) cells are highly migratory cells that contribute to various vertebrate tissues, and whose migratory behaviors resemble cancer cell migration and invasion. Information exchange via dynamic NC cell–cell contact is one mechanism by which the directionality of migrating NC cells is controlled. One transmembrane protein that is most likely involved in this process is protein tyrosine kinase 7 (PTK7), an evolutionary conserved Wnt co-receptor that is expressed in cranial NC cells and several tumor cells. In *Xenopus*, Ptk7 is required for NC migration. In this study, we show that the Ptk7 protein is dynamically localized at cell–cell contact zones of migrating *Xenopus* NC cells and required for contact inhibition of locomotion (CIL). Using deletion constructs of Ptk7, we determined that the extracellular immunoglobulin domains of Ptk7 are important for its transient accumulation and that they mediate homophilic binding. Conversely, we found that ectopic expression of Ptk7 in non-NC cells was able to prevent NC cell invasion. However, deletion of the extracellular domains of Ptk7 abolished this effect. Thus, Ptk7 is sufficient at protecting non-NC tissue from NC cell invasion, suggesting a common role of PTK7 in contact inhibition, cell invasion, and tissue integrity.

## 1. Introduction

The neural crest (NC) is an excellent model system used to study signaling mechanisms that guide cell migration. They are multipotent cells migrating over large distances throughout the embryo, giving rise to a plethora of derivatives, including neurons, glia cells, smooth muscles, and pigment cells. After undergoing an epithelial to mesenchymal transition (EMT), cranial NC cells migrate first, as a cell collective, and separate later into single migrating cells. The directionality of migrating NC cells is orchestrated by collective chemotaxis, co-attraction between collectively migrating cells, and cell contact-mediated signaling, including mechanical signals (reviewed in [1,2,3]).

Mechanisms of NC migration have been likened to cancer cell invasion [4,5], as both processes involve EMT, whereby cells lose their apical-basal polarity and experience a similar change in the composition of cell adhesion molecules. Multiple pathways and transcription factors, such as SNAIL and TWIST mediate this transition in NC and cancer cells [6,7]. Furthermore, both cell types express matrix metalloproteases to enable cells to adopt an invasive phenotype. This motile phenotype is also characterized by planar cell polarity (PCP) [8], which is regulated by a non-canonical Wnt signaling pathway. In this context, it was shown that PCP signaling plays a role in contact inhibition of locomotion (CIL), a phenomenon whereby cells change their directionality after cell–cell contact [9,10]. CIL can take place between the same types of cells (homotypic CIL) or between different cell types (heterotypic). During CIL, cells collide, establish cell–cell adhesion at the contact zone, and then retract their protrusions facing in the direction of the cell contact. Subsequently, the cells form protrusions pointing away from the contact, and separate, thereby migrating into opposite directions. While homotypic CIL serves as the driving force of directional migration of NC cells [11] and likely also cancer cells, loss of heterotypic CIL between cancer cells and healthy cells has been associated with metastasis [9,12]. Regulators of PCP signaling, such as the phosphoprotein disheveled (Dvl), have been shown to localize at cell–cell contact sites, thereby affecting the activity of small GTPases of the Rho family and, subsequently, the directionality of NC cells [8,13]. Similar mechanisms are also evident in cancer cells, underlining the similarity between NC and cancer cell migration [14,15].

A regulator of PCP signaling that has been implicated in cancer progression as well as NC migration is protein tyrosine kinase 7 (PTK7). PTK7 is an evolutionary conserved transmembrane protein with seven extracellular immunoglobulin-like domains and an intracellular kinase homology domain, which lacks catalytic activity [16,17]. Originally, PTK7 was identified as a gene upregulated in colon carcinoma cells and accordingly named colon-carcinoma kinase 4 (CCK4) [18]. As PTK7 expression is deregulated in a variety of cancer types, a function of PTK7 in cancer is likely [19]. In addition, it was shown that PTK7 plays a role in diverse developmental and physiological processes, including cell migration and invasion (reviewed in [19,20,21]). Indeed, we showed that Ptk7 is required for NC migration. Ptk7 loss-of-function inhibits cranial NC migration in *Xenopus* embryos [22] and Ptk7 morphant NC cells adopt a blebbing phenotype [23]. Furthermore, we characterized PTK7 as a regulator of planar cell polarity [24] and showed that it functions as a Wnt co-receptor recruiting Dvl to the plasma membrane [22,25,26]. Analyzing PTK7 localization in breast cancer cells, we discovered a Wnt-mediated mechanism by which PTK7 is removed from the membrane by caveolin-mediated endocytosis [27]. This process is dependent on Frizzled7, but not on Ror2 [27], which are both co-receptors of PTK7 [23,25,28]. However, it remains unclear if PTK7 is also dynamically localized in migrating NC cells and if its subcellular localization is functionally relevant. 

Here, we provide evidence that Ptk7 is dynamically localized at NC cell–cell contact sites and required for CIL. Our data suggest that the extracellular domains of Ptk7 are relevant for this localization and mediate homophilic interaction. Interestingly, these domains also seem to be relevant for protecting tissues from NC cell invasion, as overexpression of Ptk7, but not its extracellular deletion mutant, abolished NC cell invasion of non-NC tissue. Taken together, our findings provide novel insights into how migrating NC cells communicate via dynamic cell–cell contact sites and suggest PTK7 as a therapeutic target of cell invasion. 

## 2. Results

### 2.1. Ptk7 Is Dynamically Localized at NC Cell–Cell Contacts

**Figure 1:**
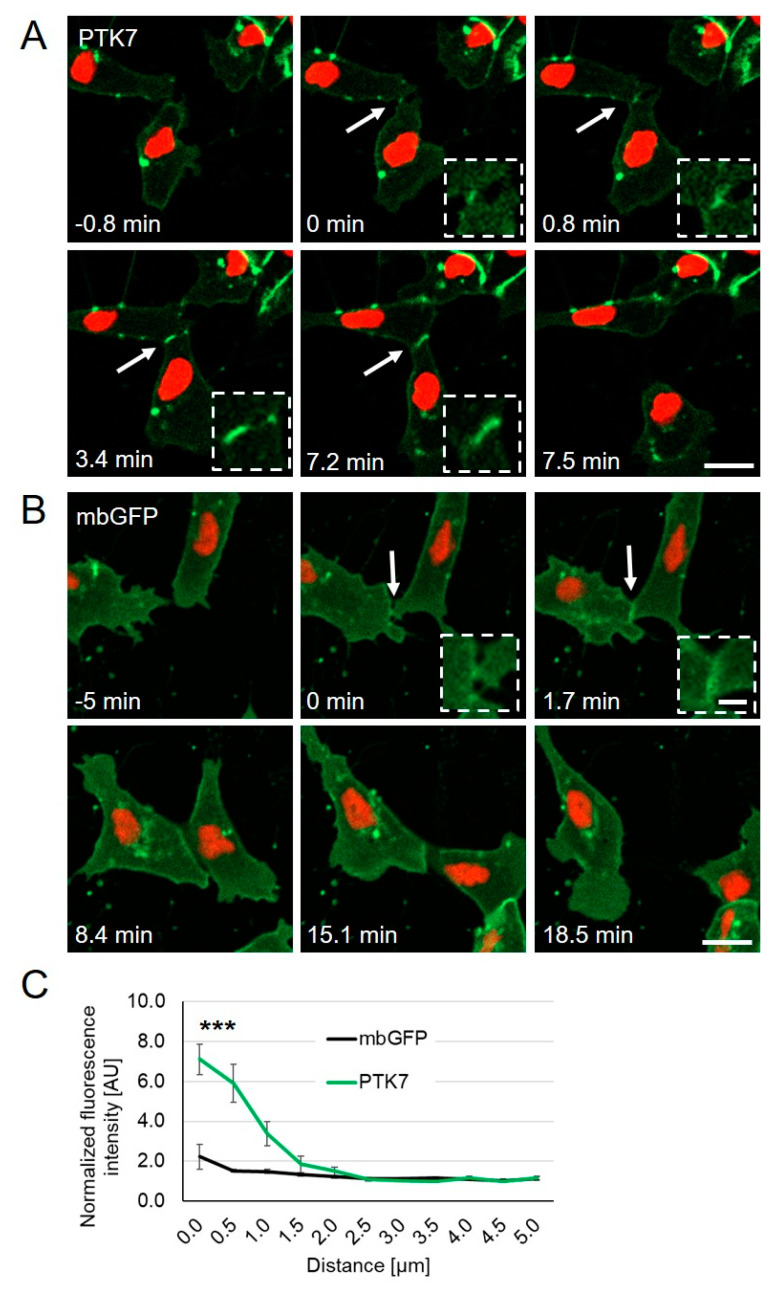
PTK7-GFP accumulates at cell–cell contact sites; *Xenopus* embryos were injected with 250 pg *PTK7-GFP* or 50 pg *mbGFP* RNA in combination with 250 pg *H2B-mcherry* RNA into one blastomere at the two-cell stage. NC cells were explanted at stage 17 and analyzed by spinning disk microscopy. (**A**) Time lapse images of PTK7-GFP expressing NC cells before (–0.8 min), during (0–7.2 min), and after (7.5 min) cell collision. The dashed square shows a higher magnification of the NC cell–cell contact zone. PTK7-GFP is highly enriched at the contact zone between NC cells, but immediately disappears when the cell–cell contacts are broken. NC cell–cell contacts are indicated by white arrows and are shown at higher magnifications in the boxed areas. Scale bar = 20 µm or 5 µm for higher magnification. (**B**) Time lapse images of mbGFP expressing NC cells before (–5 min), during (0–15.1 min) and after (18.5 min) collision. Like PTK7-GFP, mbGFP is localized at the membrane, however, it does not accumulate at cell–cell contact sites of colliding NC cells. (**C**) The graph shows the fluorescence intensity at the cell–cell contact site normalized to the membrane fluorescence at a non-contact site at 0 to 5 µm distance from the membrane. Average values and standard errors of the mean (SEM) are shown for 18 PTK7-GFP and 22 mbGFP expressing NC cells of at least three independent experiments, *** *p* < 0.005 (two-way ANOVA).

To analyze the dynamic localization of Ptk7 in *Xenopus* cranial NC cells, we expressed *GFP-tagged ptk7* RNA and monitored protein localization by live-cell imaging. *Xenopus* embryos were injected with *PTK7-GFP* RNA in combination with RNA coding for *H2B-mcherrry* to track the nuclei of migrating NC cells. *Membrane localized GFP* (*mbGFP*) was expressed as a control. NC explants were dissected at premigratory NC cell stages, cultured on fibronectin, and NC cell migration was analyzed by time lapse imaging using spinning disk microscopy. The Ptk7 protein as well as mbGFP were visible at the cell membrane of migrating NC cells (Figure 1). Interestingly, Ptk7 significantly accumulated at cell–cell contact sites of NC cells (Figure 1A, Video S1), while this was not observed for the mbGFP controls (Figure 1B, Video S2). Ptk7 clustering appeared to be transient and disappeared immediately after the cell–cell contacts were broken (Figure 1A, 7.5 min). To quantify Ptk7 accumulation at cell–cell contact sites, we determined the normalized fluorescence intensity at the time point of maximal cell–cell contact over a linear range of 0 to 5 µm, starting from the membrane and extending into the cytoplasm (Figure 1C, supplementary Figure S1A). Consistently, the Ptk7 signal at the cell–cell contact was significantly increased compared to the mbGFP controls (Figure 1C), confirming that Ptk7 transiently accumulates at cell–cell contact sites of NC cells.

### 2.2. The Extracellular Domains of Ptk7 Are Essential for Its Accumulation at Cell–Cell Contacts

**Figure 2:**
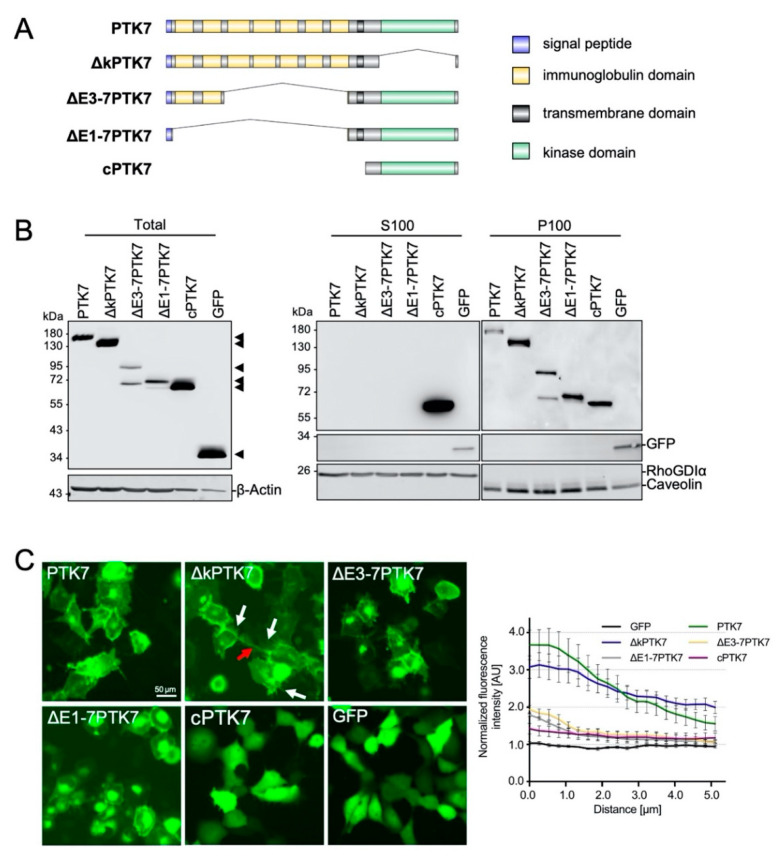
Localization of distinct Ptk7 deletion constructs in H1299 cells. (**A**) Schematic presentation of the Ptk7 domain structure and its different deletion mutants. (**B**) Transient expression of Ptk7-GFP variants in H1299 cells 24 h after transfection. Total cell lysates (left panel) were prepared by homogenizing the cells in RIPA-buffer and 50 µg of cell lysates were subjected to SDS-PAGE and immunoblotting. Due to the high expression level, 10 µg of lysate from cPTK7- and 5 µg from GFP-expressing cells were loaded. For subcellular localization (right panels) lysates were separated by 100,000× *g* centrifugation in soluble (S100) and particulate (P100) fractions. Aliquots of each fraction were analyzed by immunoblotting. GFP-containing proteins were detected by GFP-HRP antibody and ECL. Detection of β-Actin served as loading control, RhoGDIα as a marker for the cytosolic (S100) and Caveolin for the particulate (P100) fraction. (**C**) Fluorescence analysis of living Ptk7-GFP-expressing H1299 cells 24 h after transfection showing the subcellular localization of the transiently expressed GFP fusion proteins. Red arrows show tube-like structures, white arrows show membrane-spikes. Bar: 50 µm. The graph shows the fluorescence intensity at the cell–cell contact site normalized to the membrane fluorescence at a non-contact site at 0 to 5 µm distance from the membrane (mean ± SEM, *n* = 8−10).

To determine which Ptk7 domains are responsible for Ptk7 accumulation at NC cell–cell contact sites, we aimed to analyze the subcellular protein localization of different Ptk7 deletion constructs. As Ptk7 is a single-pass transmembrane protein with distinct domains, we constructed several GFP-tagged deletion constructs to investigate their effects on protein localization. In addition to full-length PTK7-GFP, deletion mutants of the kinase domain (∆kPTK7), of the extracellular domains E3–E7 (∆E3-7PTK7) and E1–E7 (∆E1-7PTK7), as well as a construct consisting only of the intracellular domain (cPTK7) were generated (Figure 2A). First, we analyzed protein localization in H1299 lung carcinoma cells. Wild type Ptk7 and all deletion mutants were detectable 24 h after transfection, although to various degrees (Figure 2B, left panel). The cPTK7 variant, comprising only the intracellular domain of the protein, was more strongly expressed than the wild type protein. In contrast, deletion of the immunoglobulin domains reduced the amount of expressed proteins. Densitometric analyses revealed for cPTK7 a 4.64 ± 1.46-fold and for ΔkPTK7 a 1.72 ± 0.35 increase compared to PTK7, and a reduction with 0.81 ± 0.22 for ΔE3-7PTK7 and 0.28 ± 0.09 for ΔE1-7PTK7 (mean ± SEM, *n* = 5). All Ptk7 variants were found in the particulate, membrane containing P100 fraction, except for cPTK7, which was highly enriched in the soluble, cytoplasmic S100 fraction (Figure 2B, right panel). Similar results were obtained when we transiently transfected HEK293 cells with the Ptk7 constructs (data not shown). The distinct localization of the Ptk7 mutants in H1299 cells was confirmed by live-cell imaging analysis (Figure 2C), showing that full-length Ptk7 as well as the variant lacking the kinase homology domain (ΔkPTK7) localized to the plasma membrane and at cell–cell contact sites. Note that ΔkPTK7 induced the formation of prominent, long membrane spikes (marked by white arrows) and tube-like structures connecting adjacent cells (red arrow). ΔE3-7PTK7 and ΔE1-7PTK7 were found at the plasma membrane and in intracellular compartments. As already seen in the fractionation experiments, cPTK7 was located in the cytoplasm and the nucleus, comparable to GFP. To determine the localization of the respective Ptk7 constructs at cell–cell contact sites of H1299 cells, we measured the normalized fluorescence intensity at cell–cell contact sites over a linear range of 0 to 5 µm starting from the membrane and extending into the cytoplasm. We find that PTK7 and ∆kPTK7 are enriched at cell–cell contact sites of H1299 cells, but not the extracellular deletion constructs, ΔE3-7PTK7, and ΔE1-7PTK7. In conclusion, these results suggest that the extracellular domains of Ptk7 determine how effective the protein is localized to membranes, in particular to cell–cell contact sites.

**Figure 3:**
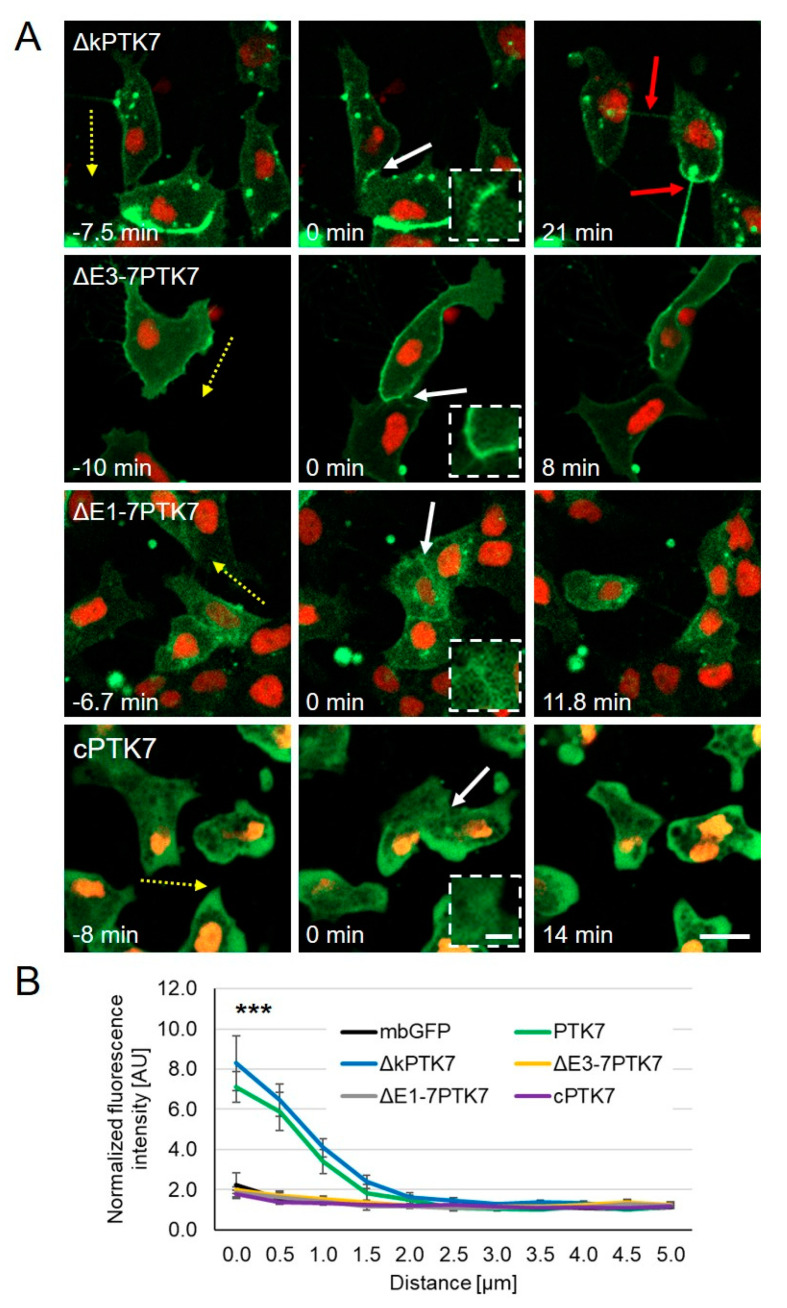
Dynamic localization of distinct Ptk7 deletion constructs during NC cell–cell contact. Two-cell stage *Xenopus* embryos were injected unilaterally with 200 pg *ΔkPTK7-GFP*, 200 pg *ΔE3-7PTK7-GFP*, 200 pg *ΔE1-7PTK7-GFP,* or 100 pg *cPTK7-GFP* RNA in combination with 200 pg *H2B-mcherry* RNA. NC cells were explanted at stage 16–18 and protein localization was analyzed by time lapse microscopy. (**A**) Images show NC cells before (first column), during (second column; 0 min), and after (third column) cell–cell contact. The respective constructs are indicated for each lane. ΔkPTK7-GFP expressing NC cells showed strong protein accumulation at cell–cell contact sites and often remained connected via nanotube-like structures after they separated (red arrows). ΔE3-7PTK7-GFP, ΔE1-7PTK7-GFP, and cPTK7-GFP were not enriched at cell–cell contacts. Yellow dotted arrows indicate the direction of migrating NC cells. NC cell–cell contacts are indicated by white arrows and shown at higher magnification in the boxed areas. Scale bar = 20 µm or 5 µm for higher magnification. (**B**) Graph showing the average fluorescence intensity at the cell–cell contact site normalized to control sites for NC cells expressing the indicated Ptk7 deletion constructs or mbGFP. Average values and standard errors of the mean are shown for 18 PTK7-GFP, 22 mbGFP, 28 ΔkPTK7-GFP, 22 ΔE3-7PTK7-GFP, 15 ΔE1-7PTK7-GFP, and 12 cPTK7-GFP expressing NC cells of at least three independent experiments. *** *p* < 0.005 for ΔkPTK7-GFP versus mbGFP (two-way ANOVA). The data for mbGFP and PTK7-GFP have already been shown in Figure 1B.

**Figure 4:**
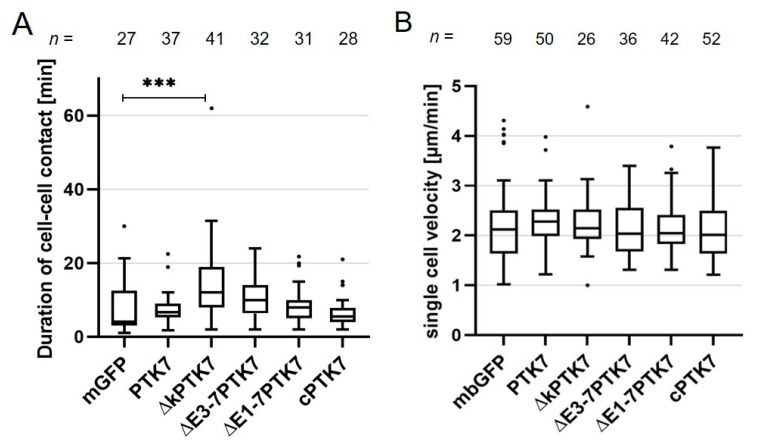
∆kPTK7 overexpressing NC cells stay longer in cell–cell contact compared to controls, while single cell velocity is not affected. (**A**) Box plot showing cell–cell contact time (in minutes) during collision of NC cells expressing various Ptk7 constructs. NC cells expressing ΔkPTK7-GFP exhibit a significantly increased contact time compared to the control. (**B**) Graph blotting single cell velocity of NC cells expressing the indicated Ptk7 constructs. Box and whiskers plots summarize results of at least three independent experiments, the number of analyzed cells for each condition is indicated at the top. The box extends from the 25th to the 75th percentile, with whiskers with maximum 1.5 IQR. The median is plotted as a line inside the box. *** *p* < 0.005 (Kruskal–Wallis test).

Next, we analyzed the dynamic protein localization of the different Ptk7 constructs in migrating *Xenopus* NC cells using time lapse imaging. Compared to wild type PTK7 (Figure 1A), ∆kPTK7 showed an even stronger accumulation at the cell–cell contact site, which seemed to persist in NC cells even after the cell–cell contacts had been dissolved (Figure 3A, Video S3). This was confirmed by analyzing the normalized fluorescence intensity at the cell–cell contact site, which showed increased values for ∆kPTK7 compared to wild type PTK7 (Figure 3B). In addition, like we previously observed in H1299 cells, the separating cells were still connected via nanotube-like structures. In contrast, deletion of the immunoglobulin domains E3–E7 in ∆E3-7PTK7 prevented protein clustering at cell–cell contact sites (Figure 3A, Video S4), although this construct was localized at NC cell membranes. Furthermore, deletion of the immunoglobulin domains E1–E7 interfered with membrane localization of the ∆E1-7PTK7 protein and the signal at the membrane was weak and did not increase at NC cell–cell contacts (Figure 3A, Video S5). Quantification of the normalized fluorescence intensities at cell–cell contact sites confirms that ∆E3-7PTK7 and ∆E1-7PTK7 are both not significantly enriched at cell–cell contact sites (Figure 3B). The Ptk7 construct containing only the intracellular domain of Ptk7, cPTK7, was expressed in the cytoplasm and nucleus and did also not show any enrichment at cell–cell contact sites (Figure 3A, Video S6), which was confirmed by measuring the normalized fluorescent intensities (Figure 3B). Furthermore, as the Ptk7 deletion constructs differentially localized in NC cells, we analyzed if they also had distinct effects on cell–cell contact duration. Indeed, we observed that ∆kPTK7 overexpressing NC cells, which showed an enhanced protein accumulation at the cell–cell contact zones, stayed longer in cell–cell contact compared to controls (mbGFP = 8.4 min ± 1.5 min, ∆kPTK7 = 15.0 min ± 1.7 min, Figure 4A). All other constructs did not significantly affect cell–cell contact time. In addition, we did also not observe any significant differences in single cell velocity between NC cells overexpressing the different Ptk7 constructs (Figure 4B). Thus, these data indicate that the kinase domain of Ptk7 plays a role in cell-contact duration, while the extracellular domains of Ptk7 seem to be essential for Ptk7 clustering at NC cell–cell contacts.

### 2.3. The Extracellular Domains of Ptk7 Mediate Homophilic Binding 

**Figure 5:**
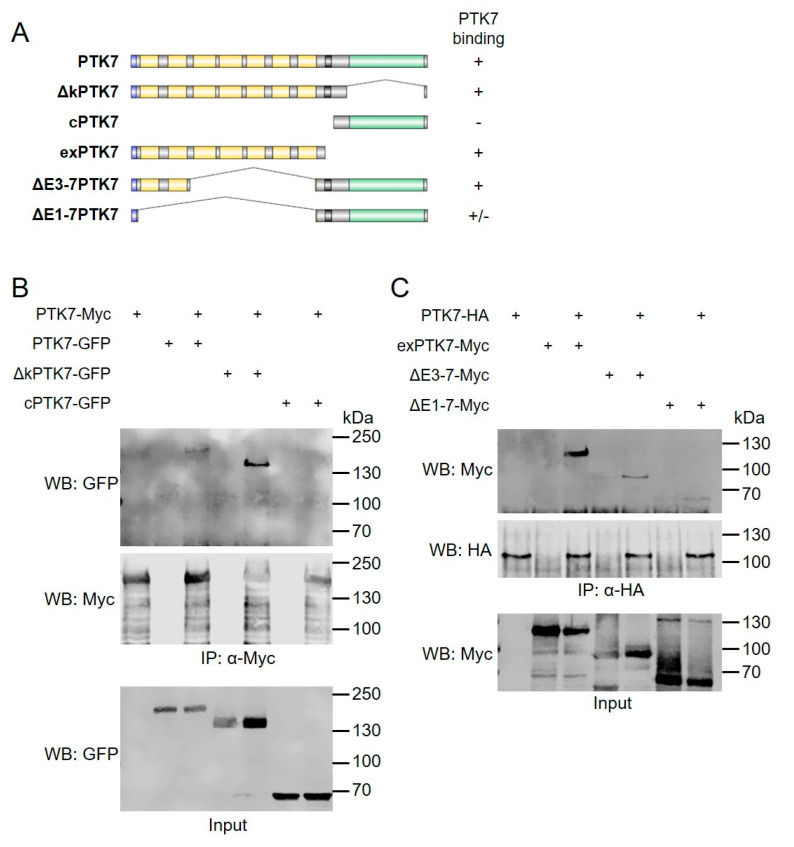
Ptk7 mediates homophilic binding via its extracellular domains. (**A**) Schematic overview of the respective constructs. Their ability to bind to full-length Ptk7 is indicated by (+), the lack of binding by (−). HEK293 cells were transfected as indicated and immunoprecipitations were performed using anti-Myc (**B**) or anti-HA antibodies (**C**). Cell lysates are shown in the bottom panel, co-immunoprecipitated proteins in the upper panel and immunoprecipitated proteins in the middle panel. Antibodies used for Western blotting (WB) are indicated on the left, molecular weights (kDa) are indicated on the right. Representative results of at least three independent experiments are shown. The quantity of PTK7 and ∆kPTK7 co-precipitated with full-length PTK7 in respect to the respective inputs was determined by densitometric analysis (LI-COR Image Studio): mean value PTK7: 1 for ∆kPTK7: 2.6.

The deletion mutants of the extracellular domains of Ptk7 do not accumulate at cell–cell contact sites of NC cells, suggesting that the extracellular immunoglobulin domains are required for Ptk7 clustering. In fact, the fly ortholog of Ptk7 has previously been shown to mediate homophilic interaction [29]. Thus, we tested the ability of the different Ptk7 deletion constructs to bind to full-length Ptk7. The different constructs are schematically shown in Figure 5A. GFP-tagged PTK7, ∆kPTK7, and cPTK7 were co-transfected with Myc-tagged PTK7 into HEK293 cells. Co-immunoprecipitations were performed using either anti-Myc (Figure 5B) or anti-GFP antibodies (Figure S2). Myc-tagged PTK7 co-precipitated PTK7-GFP as well as the kinase deletion mutant ∆kPTK7, but not the cytoplasmic PTK7 fragment, cPTK7 (Figure 5B). Conversely, PTK7-GFP and ∆kPTK7-GFP co-precipitated PTK7-myc, while cPTK7-GFP did not (Figure S2). Thus, the homophilic interaction of Ptk7 is not mediated by its intracellular domain.

To analyze the role of the extracellular domains for homophilic interaction we aimed to co-precipitate an extracellular fragment of PTK7, exPTK7, or the extracellular deletion constructs (∆E3-7PTK7 and ∆E1-7PTK7) with full-length PTK7. Indeed, HA-tagged PTK7 co-precipitated the Myc-tagged exPTK7 and vice versa (Figure 5C and Figure S2), suggesting that the extracellular domains are sufficient for homophilic binding. Deletion of the extracellular immunoglobulin domains E3–E7 did not abolish this interaction (Figure 5C and Figure S2). However, we noted that ∆E1-7PTK7 was not efficiently co-precipitated with PTK7 and it was not able to co-precipitate PTK7. As both extracellular domain deletion constructs contain the transmembrane domain, we can currently not rule out its impact on interaction. A semi-quantitative summary of the binding is shown in Figure 5A. Taken together these data show that the extracellular domains of Ptk7 are sufficient to mediate homophilic interaction, but additional domains like the transmembrane domain may also play a role.

### 2.4. Ptk7 Is Required for CIL of Migrating NC Cells

**Figure 6:**
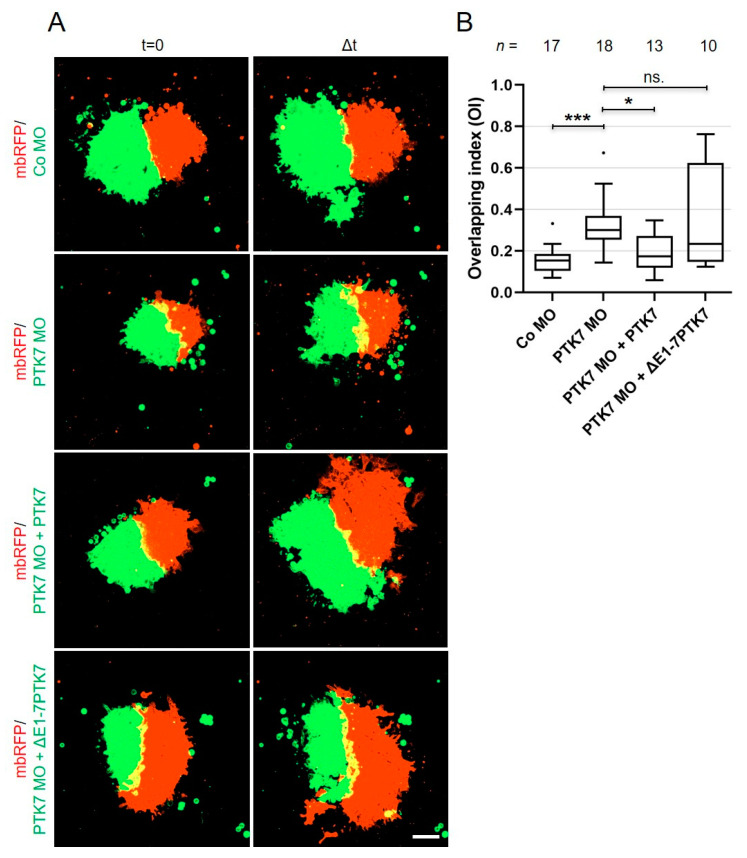
Loss of Ptk7 inhibits CIL in cranial NC explants. Confrontation assays were performed using explants injected with either 6.5 ng MO in combination with 100 pg *mbGFP* RNA or 200 pg *mbRFP* RNA as control. For rescue experiments 200 pg *PTK7* or *∆E1-7PTK7* RNA were co-injected with the MO. (**A**) First column: confronted explants at the start of the experiment (time point t = 0). Second column: confronted explants at the time point of the maximal invasion (Δt). Injected constructs are indicated for each condition on the left. The overlapping area is marked by yellow fluorescence. (**B**) Box plot showing overlapping indexes (OI) of the different NC explants with the control. The total number of analyzed confrontation events of at least three independent experiments is indicated for each column. The box extends from the 25th to the 75th percentile, with whiskers with maximum 1.5 IQR. The median is plotted as a line inside the box. * *p* < 0.05, *** *p* < 0.005, ns.: not significant (Kruskal–Wallis test).

In migrating NC cells Ptk7 dynamically clusters at cell–cell contact sites, suggesting that it may play a role in CIL. To analyze if this is the case, we performed homotypic confrontation assays [11]. Embryos were either injected with morpholino oligonucleotides (MO) in combination with *mbGFP* RNA or with *mbRFP* RNA alone. For the PTK7 MO we used a relatively low concentration to avoid the blebbing phenotype. NC explants were dissected at premigratory stages and MO-injected explants (green) were cultured in close proximity to control explants (red) on a fibronectin-coated matrix. Cell invasion was monitored using spinning disk microscopy. To measure the extent of NC cell invasion, the overlapping index (OI) comparing the overlapping area (yellow) of the confronted explants to the normalized area of one explant at the time point of maximal invasion ∆t was calculated [30]. Figure 6A shows different explants at the start of the experiment (t = 0) and at the time point of maximal invasion (∆t) (the respective experimental conditions are summarized in Video S7). As expected, control explants showed CIL and the control MO (Co MO)-injected NC cells were not invaded by the *mbRFP*-expressing control NC cells and vice versa. The respective OI was low (Figure 6B; median OI = 0.153). In contrast, NC cells injected with the PTK7 MO were invaded by the control NC cells and a significant increase in the OI compared to the control was detected (Figure 6A,B; median OI = 0.302). This effect could be rescued by co-injecting wild type *PTK7* RNA (lacking the MO binding site) with the PTK7 MO. In this case CIL was restored and the OI decreased significantly (median OI = 0.174). Interestingly, co-injection of the extracellular deletion construct *∆E1-7PTK7* was not able to rescue CIL in MO-injected NC explants (median OI = 0.248). Thus, these data suggest that Ptk7 is required for CIL and that its extracellular domains are important for this function.

### 2.5. Ptk7 Protects Non-NC Tissue from Cell Invasion 

**Figure 7:**
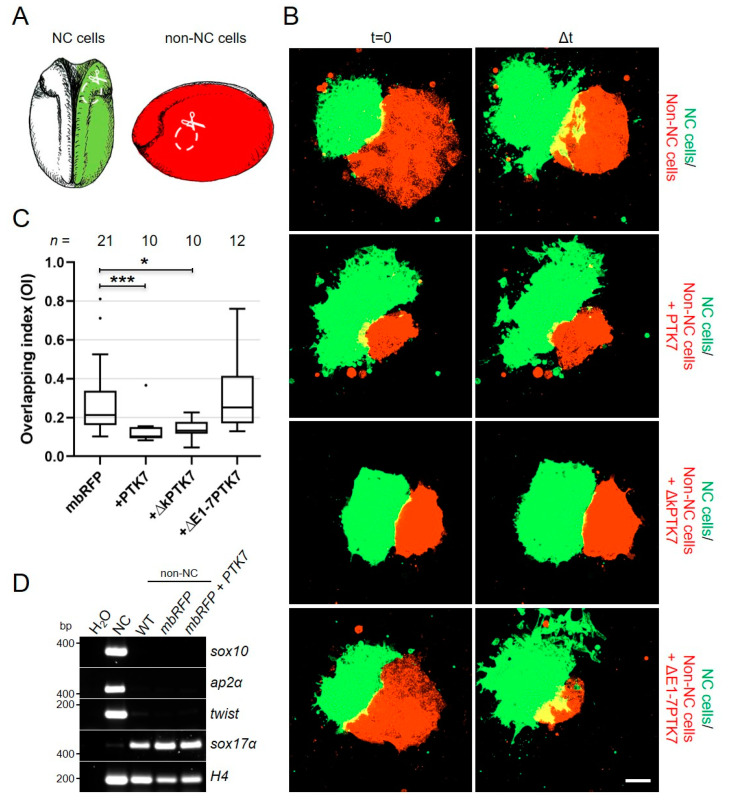
The extracellular domain of Ptk7 protects non-NC tissue from NC cell invasion. (**A**–**C**) Embryos were injected with either 100 pg *mbGFP* RNA or 200 pg *mbRFP* RNA, alone or in combination with 500 pg *PTK7, ΔkPTK7,* or *ΔE1-7PTK7* RNA, in one blastomere at the two-cell stage. (**A**) At early neurula stage, either green fluorescent NC cells or red fluorescent non-NC cells were dissected. NC cells were co-cultured with non-NC cells and analyzed by time lapse microscopy. (**B**) First column: confronted explants at the start of the experiment (time point *t* = 0). Second column: confronted explants at the time point of maximal invasion (Δ*t*). Green fluorescent NC cells invaded red fluorescent control non-NC tissue (upper lane). Overexpression of PTK7 and ΔkPTK7 in non-NC explants prevented NC cell invasion (middle lanes). Non-NC tissue expressing ΔE1-7PTK7 was invaded by NC cells (bottom panel) as it was observed in the control. Scale bar = 100 µm. (**C**) Box plot showing overlapping indexes (OI) of NC explants confronted with non-NC tissue expressing mbRFP alone or in combination with various Ptk7 deletion constructs. Overexpression of PTK7 and ΔkPTK7, but not ΔE1-7PTK7, significantly decreased the OI. Number of analyzed confrontation events of at least three independent experiments is indicated for each column. The box extends from the 25th to the 75th percentile, with whiskers with maximum 1.5 IQR. The median is plotted as a line inside the box. * *p* < 0.05, *** *p* < 0.005 (Kruskal–Wallis test). (**D**) Marker expression of the explanted tissue was determined by performing RT-PCR utilizing the NC marker *sox10, ap2α,* and *twist* as well as the endoderm marker *sox17α*. Amplification of *histone H4* (*H4*) was used as an internal control.

As Ptk7 seems to be required for CIL, we next asked if it is also sufficient for it. NC cells migrate over large distances throughout the embryo and invade a number of different tissues, while they change their direction of migration upon contact with another NC cell [11]. Thus, mechanisms are required that allow NC cells to distinguish between NC and non-NC cells. As Ptk7 dynamically localizes at NC cell–cell contacts it may contribute to this process. To examine if Ptk7 may be sufficient to protect non-NC cells from cell invasion by NC cells, we performed heterotypic confrontation assays. Embryos were either injected with *mbGFP* RNA or with *mbRFP* RNA in combination with the different Ptk7 constructs. At early neurula stages these two populations of embryos were either used to dissect GFP-marked premigratory NC cells (Figure 7A, green) or a lateral tissue piece (Figure 7A, red), which does not express NC markers as confirmed by RT-PCR (Figure 7D). Thus, for the sake of simplicity we will refer to this lateral tissue as non-NC tissue. Subsequently NC and non-NC explants were cultured in close proximity on a fibronectin-coated matrix and cell invasion was monitored using spinning disk microscopy. Figure 7B shows the co-cultures, NC cells in green and non-NC cells in red, at the start of the experiment (*t* = 0) and at the time point of maximal invasion (∆*t*); NC cells that are invading the non-NC tissue are seen in yellow (the respective experimental conditions are summarized in Video S8). To measure the extent of NC cell invasion, the overlapping index (OI) was calculated. As expected, the green fluorescent control NC cells invaded the non-NC tissue (Figure 7B) and could be traced as yellow fluorescent cells invading the non-NC tissue (red). In contrast, green fluorescent NC cells failed to invade non-NC tissue expressing PTK7 or the kinase deletion construct, ∆kPTK7, which is also reflected by a significant reduction in the OI compared to control non-NC cells (Figure 7C, control: median OI = 0.213; PTK7: median OI = 0.104, *p*-value = 0.0008; ∆kPTK7: median OI = 0.133, *p*-value = 0.0088). Interestingly, deletion of the extracellular domain abolished this protective effect and NC cells effectively invaded the non-NC cell tissue expressing ∆E1-7PTK7 and the OI was comparable to controls (∆E1-7PTK7: median OI = 0.252). Thus, ectopic expression of this construct is not able to protect non-NC cells from NC cell invasion.

One way by which ectopic expression of Ptk7 could protect non-NC cells from NC cell invasion is that it could induce expression of neural crest marker genes. To find out if this is the case, we analyzed both cell types for the expression of NC markers like *sox10, ap2α* and *twist* by RT-PCR (Figure 7D). In addition, we determined *sox17α* expression, an endodermal marker, which is expressed in the lateral tissue at the time of explantation [31,32]. NC explants as well as un-injected non-NC tissue or non-NC tissue injected with *mbRFP* or a combination of *PTK7* and *mbRFP* RNA were analyzed. NC cells expressed *sox10, ap2α* and *twist*, but not *sox17α*. In contrast, non-NC tissue was positive for *sox17α*, but expression of all NC markers was significantly reduced. The same was seen for the *PTK7*-overexpressing non-NC cells. Thus, the PCR data does not indicate that *PTK7* overexpression causes non-NC cells to adopt neural crest marker gene expression. Therefore, Ptk7 likely protects non-NC cells from invasion by a cellular mechanism involving its extracellular domain.

## 3. Discussion

Contact inhibition of locomotion is a driving force for directional migration and relevant for collectively migrating cells like NC or cancer cells. From this perspective, invasion of cells into tissues, for example, the migration of NC cells into the tissue of the pharyngeal arches or the spreading of cancer cells during metastasis, is a sign of lack of heterotypic CIL. Conversely, NC cells—and possibly also cancer cells—employ homotypic CIL to aid the dispersion of the cell collective. Therefore, analyzing the cellular as well as the molecular mechanism of CIL is critical for understanding cell movement in embryonic development as well as cancer progression. 

Ptk7 is a molecule that likely assists in CIL of collectively migrating cells. It is expressed in migrating NC cells [22] as well as in tumor cells [19] and, as we show here, localizes dynamically at cell–cell contact zones of colliding NC cells. The formation of stable cell–cell contacts between colliding cells is a critical step during CIL and likely involves cell adhesion. For example, members of the cadherin family of calcium-dependent adhesion molecules, such as Cadherin-11 or N-Cadherin, have been shown to play a role in this process [30,33,34]. Therefore, the observed clustering of Ptk7 at NC cell–cell contact zones may also be the result of an adhesive function of Ptk7. Clearly, a crucial domain of Ptk7 in mediating interaction and recognition between cells is the extracellular domain. Cells that express Ptk7 constructs with deletions of the extracellular domain do not show significant protein accumulation at cell–cell contact sites. Furthermore, these constructs fail to rescue CIL in homotypic (NC versus NC) and heterotypic (NC versus non-NC) confrontation assays. The likeliest explanation is that the extracellular IgG domains of Ptk7 mediate homophilic binding. Indeed, our data provide evidence that the extracellular part is sufficient to mediate homophilic interaction of Ptk7 molecules. Furthermore, previous findings have already shown that the fly ortholog of Ptk7, otk, mediates homophilic binding [29]. Thus, it seems likely, that homophilic interaction of Ptk7 contributes to the clustering of the protein at cell–cell contact sites thereby contributing to CIL.

We find that Ptk7 disappeared from the plasma membrane if NC cell–cell contacts are broken and returned to the membrane if new cell–cell contacts are formed. The molecular mechanism of this dynamic localization is currently unclear. In breast cancer cells, we recently discovered a caveolin-dependent mechanism by which PTK7 is removed from the membrane [27] that may also be relevant in NC cells. In the presence of canonical Wnt ligands, Ptk7 was endocytosed in a process that involved the Fz7, but not the Ror2, receptor. If this is also the case in NC cells is currently unknown. As Fz7 also localizes at cell–cell contact zones of NC cells [11], an interaction of Ptk7 with this receptor and subsequent endocytosis is possible. In breast cancer cells, we observed that the kinase deletion mutant, was—like PTK7—endocytosed in the presence of Wnt [27]. In contrast, in NC cells, ∆kPTK7 remained at the membrane even if the cell–cell contacts were dissolved, suggesting that in this setting the kinase homology domain may be relevant to rapidly remove the protein from the membrane. Previously, we and others have shown that this domain is able to interact with Dvl, RACK1 and β-catenin [22,26,35]; thus, these factors may be important for controlling PTK7 localization dynamics. In addition, factors interacting with the extracellular part of Ptk7 may be relevant for its function in CIL. In this regard, we and others have previously shown that Ptk7/otk interacts with Wnt ligands as well as Wnt receptors [23,25,28,29,36]. Thus, the extracellular domain may also be required for Wnt/receptor complex formation and possibly Dvl recruitment, which leads to local activation of RhoGTPases and is involved in CIL downstream signaling. In this context, Ptk7-mediated Dvl recruitment may also support Cadherin-11/Trio signaling, as we have recently shown that the GEF2 domain of Trio interacts with the DEP domain of Dvl [37]. Like Ptk7, Cadherin-11 is required for CIL in NC cells [30]. Cadherin-11 interacts with the RhoGEF Trio, which can activate RhoGTPases [37,38]. As Trio was also shown to be important for microtubule catastrophe at the cell–cell contact site [39], this module may provide multiple ways to relay information from the cell–cell contact site to the cytoskeleton. 

While the extracellular domain of Ptk7 seems to be relevant for NC cell–cell communication including CIL, the intracellular Ptk7 domain likely also contributes to its function in NC migration. For example, using *Xenopus* it was recently shown that C-terminal fragments of Ptk7 were able to rescue the neural tube closure defects of Ptk7 morphants [40]. As it was previously shown in fibrosarcoma HT1080 cells that an intracellular fragment of PTK7, generated through proteolysis by matrix metalloproteinases, localizes to the nucleus and leads to upregulation of Cadherin-11 [41,42] it is tempting to speculate that the intracellular domain of PTK7 may also contribute to PTK7 function in NC migration. However, so far the contribution of distinct PTK7 domains to its function in NC migration is unclear.

Our data suggest that Ptk7 is not only required, but seems to be sufficient for CIL. In fact, we find that overexpression of Ptk7 in non-NC cells is able to protect these tissues from NC invasion. These findings are also interesting in the context of tumor progression, as already Abercrombie suggested that loss of CIL towards non-transformed cells contributes to invasiveness of tumor cells [12]. Starting with the identification of *PTK7* as a gene upregulated in colon carcinoma cells, *PTK7* expression has been shown to be deregulated in a number of cancer cell types [19,20]. *PTK7* levels are high in colon, gastric, breast, prostate, and lung cancers and associated with a poor prognosis. Furthermore, studies analyzing HT1080 fibrosarcoma cells have implicated PTK7 in cancer cell motility and metastasis in fibrosarcoma [41,42]. On the other hand, it has been shown that PTK7 is downregulated in several types of cancer, including clear cell renal cell carcinoma, metastatic melanoma, pulmonary adenocarcinoma, epithelial ovarian carcinoma, esophageal squamous cell carcinoma and breast cancer cells, indicating that PTK7 can function as a tumor promotor or tumor suppressor in different tumors or organs by regulating cell proliferation, migration, invasion, or apoptosis [43,44,45]). One mechanism by which PTK7 might exert these diverse functions was discovered in esophageal squamous cell carcinoma (ESCC) cells. Ectopic expression of PTK7 increased the proliferation of PTK7-low ESCC cells, but decreased proliferation in PTK7-high ESCC cells by biphasic regulation of ERK, Akt, and Src signaling (Shin et al, 2018).

Thus far, the focus has exclusively been on PTK7 function in cancer cells and tumor progression, but not on using it as a tool to prevent cancer invasion of tumor cells with high PTK7 expression, although PTK7 has been discussed as an attractive tumor marker and therapeutic target [46,47,48]. As PTK7 overexpression protects non-NC cells from NC invasion, it is tempting to speculate that PTK7 overexpression could also block invasion of PTK7 expressing tumor cells, although in a context-dependent manner. Indeed, there is evidence that loss of heterotypic CIL may be a factor driving metastasis. For example, for metastatic prostate cancer cells, it has been shown that they invade fibroblasts dependent on EphB3 and EphB4 signaling and activation of Cdc42. CIL could be restored by knockdown of EphB3 and EphB4 [49]. Interestingly, homotypic CIL leading to the dispersal of NC cells was aided by EphA2/A4 signaling leading to the activation of RhoA [50]. While these data indicate the existence of two distinct signaling pathways to distinguish between homotypic and heterotypic CIL, our findings to Ptk7 seem to indicate that in this case a single molecule enables a CIL response—either endogenously in NC cells or ectopically when overexpressed in non-NC cells. Further research is required to dissect the molecular and cellular mechanisms by which PTK7 controls CIL and may help us to use PTK7 as a tool to target cancer invasion.

## 4. Materials and Methods

### 4.1. Constructs

Ptk7 Morpholino antisense oligonucleotides (MO) were used as published in [26]. A standard control MO from Gene Tools (Philomath, OR, USA) was used as a control. In this study, we exclusively used *Xenopus* Ptk7 constructs, in particular the following plasmids were used for RNA injection or DNA transfection: PTK7-GFP [26], PTK7-Myc [22], PTK7-HA [22], ΔkPTK7-GFP [26], ΔkPTK7-Myc [22], mbGFP [51], mbRFP [52], H2B-mcherry [38]. The cytosolic domain of PTK7 (cPTK7) was amplified by PCR using primers: 5′TTGGATCCATGGAGTGCCTC3′ and 5′CGAATCGATGGACCCTTGTGTCTTG3′, the PCR product was cut with BamHI and ClaI and inserted into pCS2+/HA. GFP-tagged cPTK7 was obtained from cPTK7-HA by removing cPTK7 with BamHI and XhoI and inserted into pCS2+/GFP. cPTK7-Myc was amplified by PCR using primers: 5′TCAGGGATCCATGGAGTGCCTC3′ and 5′CGCGATCGATGGGACCCTTGTGTCTTG3′, cut with BamHI and ClaI and inserted into pCS2+/Myc. Deletion of immunoglobulin domains (ΔE) was introduced by PCR amplification of PTK7-myc with following primers for ΔE1-E7: 5′TTGCGAGCTCAGTAAATAGGATAGCTGCTCT3′ and 5′TAATGAGCTCGCCTGTGACATCAAGCAC3′, and for ΔE3–E7: 5′ATCTGAGCTCCAGGGTGAAGTTGCGATT3′ and 5′TATCGAGCTCCTGGTCAGCTTCAAGATAGA3′, the PCR products were cut with SacI, followed by ligation. GFP-tagged ΔE1-7PTK7 and ΔE3-7PTK7 were obtained by removing the Myc-tag with ClaI and XbaI and inserting an GFP-tag.

ΔkPTK7-HA, ΔE1-7PTK7-HA and ΔE3-7PTK7-HA were amplified by PCR using ΔkPTK7-Myc, ΔE1-7PTK7-Myc or ΔE3-7PTK7-Myc as a template and the following primers: 5′TTGGATCCATGGGGCCGATTGTGCTC3′ and 5′TTCACTCGAGCTATGCGTAATCCGGTACATCGTAAGGGTATAATCGATACCCTTGTGTCTT3′, cut with BamHI and XhoI and inserted into pCS2+. The extracellular domain of PTK7 (exPTK7) with Myc was amplified by PCR using primers: 5′TTGGATCCATGGGGCCGATTGTGCTC3′ and 5′TTATCGATTCTGGATGAGTTTGTATGGGGAA3′, cut with BamHI and ClaI and inserted into pCS2+/Myc.

### 4.2. Embryo Manipulation

*Xenopus laevis* embryos were obtained by in vitro fertilization and developmental stages were defined according to [53]. Sense capped mRNA was synthesized using the SP6 mMESSAGE mMACHINE^®^ System (Thermo Fisher Scientific, Waltham, MA, USA)) according to the manufacturer’s protocol. Injections were performed into one blastomere of 2-cell stage embryos.

### 4.3. NC Cell Explants and Analysis

Explantation of NC cells was carried out as described in [54,55]. The explants were incubated in 0.8 × MBS on fibronectin-coated (10 µg/mL, F1141-5MG, Sigma Aldrich, St. Louis, MO, USA) chamber slides for at least 30 min at 14 °C to ensure adherence of the cells. Explants were imaged using the Zeiss Spinning Disk system (Axio Observer Z1 with a 10 × or 25 × oil objective). Image analysis of NC cells was performed using Zen blue, ImageJ or MATLAB software. Fluorescence intensity of PTK7 was quantified using ImageJ. Therefore, the fluorescence intensity along a straight line drawn from the cell membrane to the cytoplasm (5 µm) was measured. The fluorescence intensity was measured at the time point of maximal cell–cell contact at the cell–cell contact zone and at a control site, without protrusion or cell–cell contacts (supplementary Figure S1). Normalization was performed by dividing the fluorescence intensity at the contact site by the fluorescence intensity of the control site of a cell. 

For heterotypic confrontation assays, a non-NC tissue piece comparable to the size of the NC explant was explanted from the lateral side of the embryo (stage 16/17). NC and non-NC tissue were placed in close proximity on fibronectin-coated chamber slides and NC cell invasion was analyzed by time lapse microscopy for at least four hours. For homotypic NC confrontation assays, two NC explants were placed in close proximity. The overlapping area (yellow) at the time point of maximum invasion (Δ*t*) was measured and the overlapping index (OI) was calculated using a custom-made MATLAB script [30]. 

### 4.4. Cell Transfection and Co-Immunoprecipitation

Transfection of H1299 cells: H1299 lung carcinoma cells were transiently transfected with pCS2+/PTK7-GFP variants using Lipofectamine 2000 (Thermo Fisher Scientific, Langenselbold, Germany). H1299 cells (1 × 10^6^/100 mm dish) were transfected 24 h after seeding with 8 µg of plasmid and 25 µL of Lipofectamine 2000 solved in 500 µL Opti-MEM I (Thermo Fisher Scientific, Langenselbold, Germany). The reaction mixture was incubated for 10 min at room temperature, applied to the cells kept in 4 mL DMEM with 10% FCS and incubated for 5 h. Thereafter, the medium was exchanged and cells were incubated for an additional 24 h. Live cell images were recorded at an inverse IX81 fluorescence microscope using CellSens Dimension (1.6) software (Olympus, Hamburg, Germany). 

Subcellular fractionation: confluent cells (5 × 10^6^ cells/100 mm dish) were scraped into 250 μL HEPES buffer (50 mM HEPES, pH 7.6, 8.6% (*m*/*v*) sucrose, 10 mM EDTA, 10 mM EGTA, protease inhibitor mix) and homogenized by forcing the mixture ten times through a 0.5 × 0.25 mm needle attached to a syringe. Nuclei and whole cells were removed by centrifugation at 600× *g* for 10 min at 4 °C. Particulate, membrane containing fractions (P100) and soluble, cytosol containing fractions (S100) were separated by centrifugation at 100,000× *g* for 1 h at 4°C. The particulate fraction was washed with 250 µL of HEPES buffer at 100,000× *g* for 15 min and resuspended in 50–100 µL of RIPA buffer [50 mM Tris/HCl, pH 8.0, 150 mM NaCl, 1% (*v*/*v*) Triton X-100, 0.5% (*w*/*v*) sodium deoxycholate, 0.1% sodium dodecyl sulfate, protease inhibitor mix]. Total cell lysates were prepared by lysing the cells in RIPA buffer. Cell lysate proteins (25–50 μg) were fractionated on SDS-polyacrylamide gels and transferred onto nitrocellulose membranes. Proteins were detected using appropriate specific antibodies and visualized using the Odyssey Sa Infrared Imaging System (LI-COR Bioscience, Lincoln, NE, USA) or enhanced chemiluminescence (ECL) (Thermo Fisher Scientific, Langenselbold, Germany) and Fusion SL4-3500.WL, Chemiluminescence Imaging System Vilber Lourmat, Eberhardzell, Germany). Antibodies used in this study were commercially available: GFP-HRP (clone GG4-2C2.12.10; Miltenyi, Bergisch Gladbach, Germany), Caveolin (#610059; Becton Dickinson, Heidelberg, Germany), RhoGDIα (Κ21) (#sc-359; Santa Cruz Biotechnology, Heidelberg, Germany), β-Actin (AC-15, Sigma-Aldrich, Taufkirchen, Germany). HRP-linked secondary antibodies were purchase from New England Biolabs (Frankfurt a. Main, Germany), CF™680- or CF™770-coupled antibodies were from Biotrend (Cologne, Germany).

Cell lines and culture conditions: H1299 cells (CRL-5803; ATCC, Manassas, VA, USA) were grown in Dulbecco’s modified Eagle medium, high glucose (DMEM) supplemented with 10% fetal calf serum (FCS) (Capricorn, Ebsdorfergrund, Germany), 1% GlutaMAX and 1% non-essential amino acids (both from Gibco-Thermo Fisher, Freiburg, Germany) at 37°C in a humidified atmosphere with 10% CO_2_ and routinely tested for mycoplasma contamination. HEK293 cell transfection and co-immunoprecipitation (co-IP) were performed as described in [37] using jetPEI (Polyplus, Graffenstaden, France ) as transfection reagent and Dynabeads^TM^ Protein G Immunoprecipitation Kit (Thermo Scientific, Waltham, MA, USA) for co-IP. For co-IP the following antibodies were used: anti-HA (Covance, MMS-101P, 1:100), anti-myc (Abcam, ab19234, 1:250) and anti-GFP (Abcam, ab290, 1:250). These and the following antibodies were also used in Western blot: anti-HA (Abcam, ab9110, 1:1000), anti-Myc (Sigma, M5546, 1:2000), anti-GFP (Roche, 1181446000, 1:1000), anti-goat-800 (LI-COR, 926-32214, 1:7500), anti-goat-680 (LI-COR, 926-68074, 1:7500), anti-mouse-680 (LI-COR, 926-32212, 1:7500), anti-mouse-800 (LI-COR, 926-68072, 1:7500), anti-rabbit-680 (Li-COR, 956-68073, 1:7500), and anti-rabbit-800 (Li-COR, 926-32213, 1:7500). 

### 4.5. RNA Purification and RT-PCR

To determine marker expression of the explanted tissue by RT-PCR total RNA from 20 NC/ non-NC explants of early neurula embryos was extracted. RNA isolation was performed using the GE Healthcare illustra RNAspin Mini Isolation Kit (Chalfont St Giles, England) according to the manufacturer’s instructions. For reverse transcription, MuLV Reverse transcriptase (Thermo Fischer Scientific, Waltham, MA, USA) was used and PCR including 28 cycles was performed. The primers corresponding to *H4* [56] were used as previously described. Primers for *ap2α*, *sox10, twist* and *sox17α* were designed as follows: *ap2α* forward, 5’-CGGGTATGTGTGCGAAACAG-3’; *ap2α* reverse, 5’-GGCGGGAGACCAATAGAGAA-3’; *sox10* forward, 5’-TCACGTTAAGCGGCCAATGA-3’, *sox10* reverse, 5’-CATGGGAGAACCATGTCGGT-3’; *twist* forward, 5’-GGGATGCAGAAAGAGGCGAT-3’, *twist* reverse 5’-AAGGCTTCGTTGAGGGACTG-3’; *sox17α* forward, 5’-ATGAGCAGCCCTGAT-3’, *sox17α* reverse, 5’-CCTGTTTCCTCCTGC-3’. PCR products were analyzed by agarose gel electrophoresis and imaged using the Odyssey Fc Imaging System (LI-COR Bioscience, Lincoln, NE, USA). 

## Data Availability

The data is contained within the article or supplementary material.

## Supplementary Materials

The following are available online at https://www.mdpi.com/article/10.3390/ijms22179324/s1, Figure S1: Average fluorescence intensity at the cell-cell contact site normalized to control sites for NC cells expressing various Ptk7 deletion constructs or mbGFP. Figure S2: Immunoprecipitation using full-length Ptk7 in combination with different Ptk7 deletion constructs. Video S1: Localization of PTK7-GFP in cranial NC cells. PTK7-GFP accumulates at cell-cell contact sites of colliding NC cells but is rapidly removed after cell-cell contacts are dissolved (white arrow). Video S2: Localization of mbGFP in cranial NC cells. mbGFP is localized at the membrane but does not accumulate at cell-cell contact sites (white arrow). Video S3: Localization of ΔkPTK7-GFP in cranial NC cells. ΔkPTK7-GFP strongly accumulates at cell-cell contact sites even after the cell-cell contact is broken (white arrow). Separating cells often remain connected via nanotube-like structures (red arrow). Video S4: Localization of ΔE3-7PTK7-GFP in cranial NC cells. ΔE3-7PTK7-GFP localizes at NC cell membranes but is not enriched at cell-cell contact sites (white arrow). Video S5: Localization of ΔE1-7PTK7-GFP in cranial NC cells. ΔE1-7PTK7-GFP does not accumulate at NC cell-cell contact sites (white arrow). Video S6: Localization of cPTK7-GFP in cranial NC cells. cPTK7-GFP is expressed in the cytoplasm and is not enriched at cell-cell contact sites (white arrow). Video S7: Homotypic confrontation assay. Control NC cells (red) were confronted with NC explants injected with MO alone or in combination with *PTK7* or *ΔE1-7PTK7* RNA (green). Injection of PTK7 MO disturbed normal CIL behavior, as the overlapping area (yellow) was increased. This effect could be rescued by co-expression of *PTK7* but not by *ΔE1-7PTK7*. Video S8: Heterotypic confrontation assay. NC cells (green) were confronted with non-NC tissue injected with *mbRFP* alone or in combination with *PTK7*, *ΔE1-7PTK7* or *ΔkPTK7* RNA (red). As expected, control non-NC tissue was invaded by NC cells. However, expression of PTK7 and ΔkPTK7, but not ΔE1-7PTK7, prevented NC cell invasion.
