# Supplementary material for: Ptk7 Is Dynamically Localized at Neural Crest Cell–Cell Contact Sites and Functions in Contact Inhibition of Locomotion
Source: Int J Mol Sci. 2021 Aug 28;22(17):9324. doi: 10.3390/ijms22179324 (PMC8431534; doi:10.3390/ijms22179324)
Supplement: Supplementary file 1 [file ijms-22-09324-s001.zip › ijms-1329346-supplementary/Supplement/ijms-1329346-supplementary.pdf]

Supplementary Figure S1

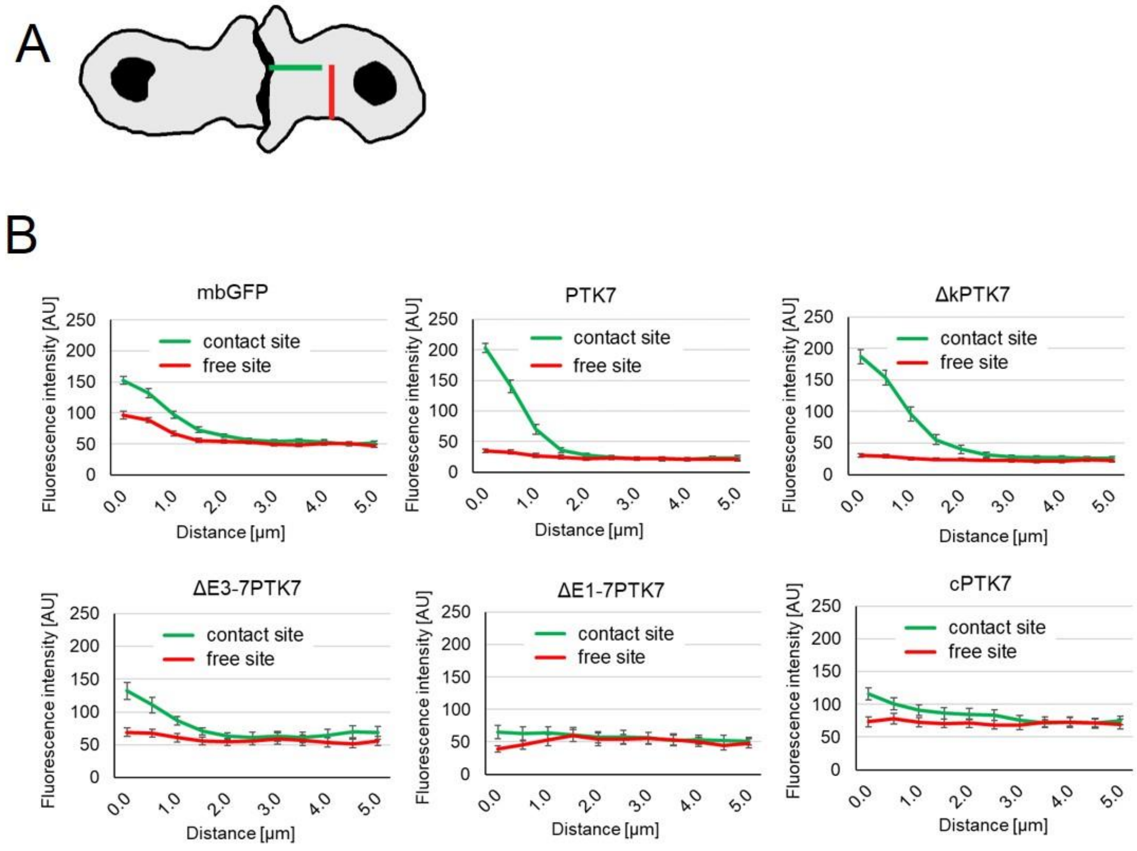

Supplementary Figure S1. Average fluorescence intensity at the cell-cell contact site normalized to control sites for NC cells expressing various Ptk7 deletion constructs or mbGFP. (**A**) Schematic representation showing two cells at the moment of maximal cell-cell contact. The green bar marks the area (5 µm) along which the fluorescence intensity at the cell-cell contact site was determined. The red bar marks the measured fluorescence intensity at a control membrane (no cell-cell contacts or protrusions). (**B**) The graph shows the fluorescence intensity at the cell-cell contact site (green) as well as the membrane fluorescence at a non-contact site (red) at 0 to 5µm distance from the membrane of NC cells expressing either mbGFP, PTK7-GFP, ΔkPTK7-GFP, ΔE3-7PTK7-GFP, ΔE1-7PTK7-GFP or cPTK7-GFP.

Supplementary Figure S2

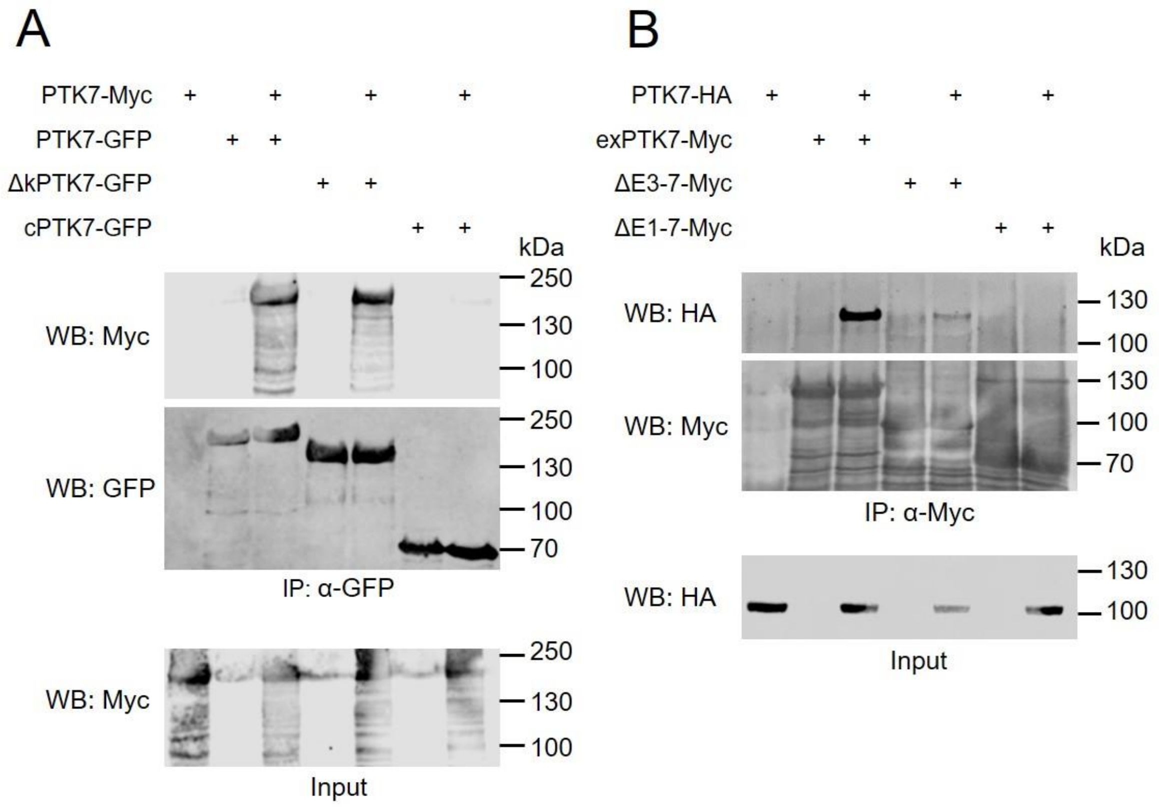

Supplementary Figure S2. Immunoprecipitation using full-length Ptk7 in combination with different Ptk7 deletion constructs. (**A**) or anti-myc antibodies (**B**) Cell lysates are shown in the bottom panel, immunoprecipitations in the upper panels. Antibodies used for Western blotting (WB) are indicated on the left, molecular weights (kDa) are indicated on the right. Representative results of at least three independent experiments are shown. The quantity of PTK7-MyC co-precipitated with PTK7-GFP or ΔPTK7-GFP in respect to the input was determined by densitometric analysis (Licor Image Studio): means were 1 for PTK7-Myc co-precipitated with PTK7-GFP and 0.87 for PTK7-Myc co-precipitated with ΔkPTK7-GFP.
